# Elevated Arsenic and Uranium Concentrations in Unregulated Water Sources on the Navajo Nation, USA

**DOI:** 10.1007/s12403-016-0226-6

**Published:** 2016-08-23

**Authors:** Joseph Hoover, Melissa Gonzales, Chris Shuey, Yolanda Barney, Johnnye Lewis

**Affiliations:** 1 Community Environmental Health Program, College Of Pharmacy, University of New Mexico, 1 University of New Mexico, Albuquerque, NM 87131 USA; 2 Department of Internal Medicine, School of Medicine, University of New Mexico, 1 University of New Mexico, Albuquerque, NM 87131 USA; 3 Southwest Research and Information Center, 105 Stanford Drive SE, Albuquerque, NM 87106 USA; 4 Navajo Nation Environmental Protection Agency - Public Water Systems Supervisory Program, PO Box 339, Window Rock, AZ 86515 USA

**Keywords:** Unregulated water sources, Arsenic, Uranium, Inorganic chemical mixtures

## Abstract

Regional water pollution and use of unregulated water sources can be an important mixed metals exposure pathway for rural populations located in areas with limited water infrastructure and an extensive mining history. Using censored data analysis and mapping techniques we analyzed the joint geospatial distribution of arsenic and uranium in unregulated water sources throughout the Navajo Nation, where over 500 abandoned uranium mine sites are located in the rural southwestern United States. Results indicated that arsenic and uranium concentrations exceeded national drinking water standards in 15.1 % (arsenic) and 12.8 % (uranium) of tested water sources. Unregulated sources in close proximity (i.e., within 6 km) to abandoned uranium mines yielded significantly higher concentrations of arsenic or uranium than more distant sources. The demonstrated regional trends for potential co-exposure to these chemicals have implications for public policy and future research. Specifically, to generate solutions that reduce human exposure to water pollution from unregulated sources in rural areas, the potential for co-exposure to arsenic and uranium requires expanded documentation and examination. Recommendations for prioritizing policy and research decisions related to the documentation of existing health exposures and risk reduction strategies are also provided.

## Introduction

Rural and tribal populations are known to experience greater health disparities than other groups in the United States (Jones 2006). The Indian Health Services has compiled data that indicates higher rates of infectious disease mortality, diabetes, liver disease, and birth defects among Native groups (Indian Health Service 2015). Chemical exposure from environmental sources, such as abandoned hardrock mines, may be one of several factors (e.g., health care access, socio-economic status) contributing to these disparities (Lewis et al. 2015). There are more than 160,000 abandoned hardrock mines in the western United States, which are frequently located on tribal lands (Government Accountability Office 2011), and are known to contain a variety of deleterious chemicals including arsenic (As) and uranium (U) (Blake et al. 2015).

People may be exposed to environmental chemicals via water ingestion, which is concerning because tribal populations are disproportionally impacted by health-based water quality violations and lack of water infrastructure (Government Accountability Office 2011; Indian Health Service 2012; Leeper 2003; VanDerslice 2011). Nationally, 12 % of tribal public water systems have health based violations, compared to 6 % of non-tribal water systems (US EPA 2015). Additionally, between 7.5 and 12 % of Native households lack access to a public water system and for some tribes, such as Navajo, as much as 30 % of households lack public water access. People with limited water infrastructure have greater reliance on unregulated water sources, which unlike public water systems, are infrequently, if ever, tested for metals or other toxicants (Jones et al. 2006; Simpson 2004). Consequently, in rural and underserved areas with limited access to regulated drinking water sources, water users may be unaware that consuming water from an unregulated source poses potential health risks due to chemical exposure (Backer and Tosta 2011; Shrivastava 2015).

**Fig. 1:**
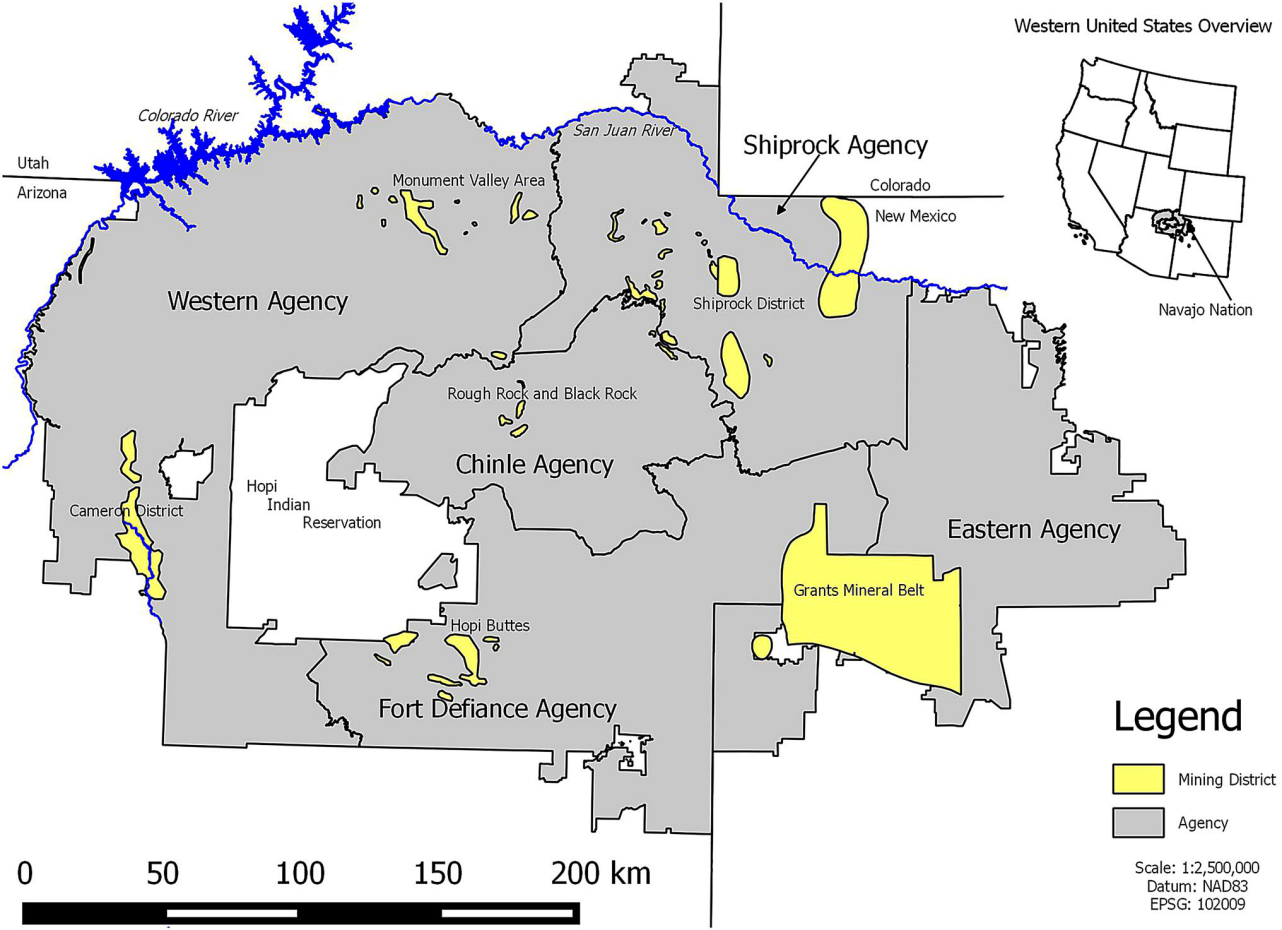
*Overview map* of the Navajo Nation, in the southwest United States, including management agencies and historic mining areas

The tribal lands of the Navajo Nation (Navajo), located in the southwestern United States (Fig. 1), are sparsely populated, with a large proportion of the population living in rural and geographically remote locations with limited water infrastructure. Approximately 30 % of Navajo households lack access to a public water system. Additionally, there are community concerns about water quality impacts from the 521 abandoned uranium mines located throughout Navajo. Between 1942 and 1989 more than 50,000 metric tons of uranium oxide (U_3_O_8_) was extracted from U mines on Navajo (US EPA 2006), including mines in the Grants Mineral Belt and Shiprock Uranium District in New Mexico and northeast Arizona (McLemore 2011), Monument Valley along the Arizona/Utah border (Chenoweth and Malan 1973), and the Cameron Area of western Navajo (Chenoweth 1993) (Fig. 1). The legacy of Cold War uranium mining on Navajo left hundreds of abandoned and unreclaimed surface, portal and vertical mine sites (US EPA 2006), and waste piles known to contain As and U (Blake et al. 2015).

Documented use of unregulated water sources, naturally occurring mineral deposits, and environmental perturbation from historic mining creates significant uncertainty regarding the potential for human exposure to As and U. Previous work investigating water quality of unregulated sources on Navajo, and the western United States more generally, has indicated the occurrence of As and U (Camacho et al. 2011; deLemos et al. 2009; US EPA 2006). While previous work has documented the occurrence of these chemicals individually, co-occurrence was not evaluated, and the occurrence of these chemicals relative to abandoned mines was not evaluated. Furthermore, since the Navajo Nation encompasses 70,000 square kilometers in the southwest United States; a geospatial evaluation of As and U occurrence is necessary to discern spatial variability for prioritizing public policy goals to improve safe drinking water access and address community concerns regarding chemical exposure.

This study is situated at the intersection of human health and rural water quality. Two research questions were addressed: (1) What is the spatial distribution, occurrence, and co-occurrence of arsenic and uranium; and (2) How does arsenic and uranium occurrence vary with proximity to an abandoned mine site? Using censored data analysis and mapping techniques we evaluated the geospatial distribution of arsenic and uranium in more than 460 unregulated water sources. We comment on the policy implications for improving public water access, possible remediation strategies, and potential human health impacts of co-exposure.

## Methods

### Study Area

The present study is limited to the boundaries of the Navajo Nation (Fig. 1) spanning 70,000 square kilometers across the Four Corners area of New Mexico, Arizona and Utah. Politically, Navajo is comprised of 110 tribal Chapters and five management agencies: Chinle, Eastern, Fort Defiance, Shiprock and Western Agencies. According to the 2010 US Census, 173,667 people live on the Navajo Nation (90 % are Navajo). Elevation ranges between 900 and 3000 meters and precipitation ranges between 80 and 1000 mm annually.

### Water Quality Datasets and Data Management

Water quality information for unregulated sources were combined from water quality surveys conducted over a 25 year period by tribal, federal and academic entities, which characterized water quality for a portion of unregulated sources throughout Navajo (Table 1). To date, these data have never been compiled and collectively analyzed. These surveys included those conducted by the US Army Corps of Engineers (US EPA 2000a; US EPA 2006), US Geological Survey (US EPA 2006), the Diné Network for Environmental Health (DiNEH) Project (deLemos et al. 2009), the Church Rock Uranium Monitoring Project (Shuey 2007), and water quality sampling conducted by the Navajo Nation Environmental Protection Agency (NNEPA), the US Centers for Disease Control and Prevention (CDC) and US EPA Region 9 between 2006 and 2010 (US EPA Region IX 2008; US EPA Region IX 2010; US EPA Region IX 2011). The water sources sampled during these surveys were selected because people hauled water from them (or the sources were thought to be used for water hauling) and there were concerns by community members about the chemical quality of the water.

**Table 1:** Previous water quality surveys of unregulated water sources on the Navajo Nation

Water quality survey	Lead agency	Sampling area (management agency)	Years	UWSs sampled	Reporting limit	
					Arsenic	Uranium
Abandoned Uranium Mines Project^a^	US EPA, US Army Corps of Engineers^o,p^	Chinle, Fort Defiance, Shiprock and Western	1993–2000	183	10 µg/L^h^	Variable pCi/L^i^
Briet Sampling^b^	US Geological Survey^q^	Fort Defiance	2001–2004	18	1 µg/L^j^	0.01 µg/L^j^
DiNEH Project^c^	University of New Mexico, Southwest Research and Information Center, Diné Network for Environmental Health^r,s,t,u,v,w^	Eastern	2003–2010	97	5 µg/L^h,k,l,m^	0.25 µg/L^k,l^
Navajo Nation Unregulated Water Source Sampling^d^	Navajo Nation EPA and Centers for Disease Control and Prevention^x^	Chinle, Fort Defiance, Shiprock and Western	2006–2007	215	1 µg/L^l^	1 µg/L^l^
Navajo Nation Drinking Water Source Sampling^e^	US EPA Region 9^s^	Eastern	2008	48	1 µg/L^l^	0.5 µg/L^l^
Radiation Assessment of Unregulated Drinking Water Sources^f^	US EPA Region 9^u^	Eastern	2009	32	5 µg/L^l^	1 pCi/L^n^
Navajo Water Well Sampling^g^	US EPA Region 9^u^	Eastern	2010	11	5 µg/L^l^	1 pCi/L^n^

^a^ US EPA (2000a); ^b^ US EPA (2006); ^c^ Unpublished project data and deLemos et al. (2009); ^d^ Unpublished project data and Murphy et al. (2009); ^e^ US EPA Region IX (2008); ^f^ US EPA Region IX (2010); ^g^ US EPA Region IX (2011)

Analysis Methods: ^h^ US EPA (1994); ^i^ Krieger and Whittaker (1980); ^j^ USGS (2002); ^k^ US EPA (2007a); ^l^ US EPA (2007b); ^m^ US EPA (1996); ^n^ US DOE (2000)

Laboratory: ^o^ Quanterra Environmental Laboratories; ^p^ Missouri River Laboratory; ^q^ NationalWater Quality Laboratory; ^r^ Carlsbad Environmental Monitoring & Research Center; ^s^ US EPA Region IX Laboratory; ^t^ US EPA Radiation and Indoor Environments National Laboratory; ^u^ GEL Laboratories; ^v^ New Mexico Scientific Laboratory Division; ^w^ Navajo Tribal Utility Authority Laboratory; ^x^ Colorado Department of Public Health and Environment Laboratory

The water quality data were compiled into a PostgreSQL relational database (version 9.3.5 (64 bit)) with PostGIS (version 2.1.3). The combined dataset contained water quality data for a total of 468 distinct unregulated sources located within the boundaries of Navajo. The variety of source types (i.e., water wells, springs and storage tanks), terrain, and geology provide a broad representation of the distribution of inorganic chemicals in unregulated sources available to Navajo residents.

Unregulated sources were flushed for 1–2 min prior to sample collection. This was done so that water samples were representative of the water quality characteristics that Navajo residents who haul water from these sources would encounter (US EPA Region IX 2008). Samples were preserved with nitric acid prior to analysis. All samples were analyzed for dissolved As by certified drinking water laboratories using US EPA analysis method 200.7/6010B/ILMO 3.0 (Inductively Coupled Plasma-Atomic Emissions Spectroscopy) or 200.8/ILMO 3.0 (Inductively Coupled Plasma-Mass Spectroscopy). Uranium activity (pCi/L) was determined using US EPA method 907.0 or HASL 300 U-02-RC and dissolved U (μg/L) was determined using US EPA method 200.7 or 200.8. Specific laboratories utilized for each dataset are noted in Table 1. A small subset of samples (13 sources) from the DiNEH Project were analyzed for As and U by the Carlsbad Environmental Monitoring and Research Center, which is not a certified drinking water laboratory.

### Water Quality Data Selection

For comparison to the uranium Safe Drinking Water Act Maximum Contaminant Level (MCL) of 30 µg/L, we converted U activity measurements to U mass using the assumption that 1 µg U was equivalent to 0.67 pCi of U activity (US EPA 2000b; Weiner 2013). Water quality results without available documentation of laboratory analysis method, or censoring (reporting limit) levels were excluded from the analysis. If multiple results were available for an unregulated source (approximately 20 % of sampled sources) the maximum observation was selected for analysis so that we would capture all possible water sources, and potential exposure sources, with national drinking water standard exceedances. Samples collected from 464 unregulated sources met these inclusion criteria. Results from 4 water sources were excluded because of insufficient information about laboratory analysis methods or data censoring (i.e., failure to state the limit of detection (LOD) when results were reported to be less than LOD).

### Statistical Analysis

Based on the marginal distribution of the data and the occurrence of multiple censoring levels (due to analysis by laboratories using several methods), the semi-parametric “Robust” Regression on Order (R-ROS) method was used to generate summary statistics. This method has previously been applied to water quality data with multiple censoring levels (Helsel 2005; Lee and Helsel 2005; Levitan et al. 2014). Using the R-ROS method the Weibull plotting position of the censored and uncensored observations are determined. Next, a linear regression is created using the plotting positions and the normal scores of the uncensored observations. Subsequently, values for censored observations are estimated using the regression model. Lastly, the estimated censored observations are combined with the uncensored observations and summary statistics are calculated. The distribution of the original As and U observations were log-normally distributed so all observations were log transformed prior to implementing the R-ROS method; the summary statistics were then retransformed to the original units. Including estimates for censored observations with uncensored observations has been shown to reduce power transformation bias (Helsel 2012; Lee and Helsel 2005). Correlation between As and U was determined using Kendall’s Tau (*τ*).

Additionally, we determined the frequency that sources exceeded the As or U MCL and the frequency at which sources exceeded *both* MCLs. The current MCL for As is 10 µg/L and for U 30 µg/L. Because the toxicity for mixtures of As and U in drinking water is unknown, we also determined the frequency of sources that produce water with As and U concentrations that exceed half of their respective MCL. Subsequently, we geographically subset the analytical results by management agency (defined previously in Sect. 2.1) and calculated agency specific measures of centrality and MCL exceedance frequency. Lastly, we calculated summary statistics based on distance between water sources and abandoned U mines, including splitting the sources into two groups using a threshold of 6.4 km (4 miles). Abandoned uranium mine locations were determined by the US Environmental Protection Agency (US EPA 2000a; US EPA 2006). We selected this distance because it was previously used by the US EPA in hazard ranking system for prioritizing abandoned mine reclamation on Navajo (US EPA 2006). All statistical analyses were completed using the NADA package (Lee 2013) for R version 3.1.1 (R Core Team 2014).

### Geospatial Distribution

The latitude and longitude of each water source, recorded using a global positioning system (GPS) at the time of sample collection, was used to map As and U concentrations for each source. The geospatial distribution of inorganic chemical concentrations was evaluated visually to identify localized areas of potentially high exposure to either As or U individually or co-exposures to these chemicals. All maps were created using QGIS 2.4.0.

## Results

Arsenic and U concentrations for Navajo overall and by geographic region are shown in Table 2. Approximately half of the tested sources had detectable concentrations of As (median 2.0 µg/L) of which 15.1 % exceeded the As MCL. A majority (75 %) of sources had detectable concentrations of U (median 3.8 µg/L) of which 12.5 % exceeded the U MCL. The distributions of As and U differed by geographic area. Arsenic was detected in more than one-third of sources in Chinle Agency, compared to 70.1 % in Fort Defiance Agency. Uranium was detected in almost half of sources in Eastern Agency compared to 95.5 % in the Western Agency. MCL exceedances for As and U followed the same spatial pattern at the Agency level.

**Table 2:** Detection frequency, median concentration and MCL exceedance frequency for As and U

	*N* ^a^	Detection frequency (%)	Median (µg/L)	UWSs exceeding MCL (%)
Arsenic				
Navajo Nation	463	55.1	2.0	15.1
*Chinle*	89	34.8	0.7	8.0
*Eastern*	87	43.7	0.3	8.5
*Fort Defiance*	97	70.1	3.6	26.0
*Shiprock*	79	57.0	3.0	14.5
*Western*	111	65.8	4.0	19.0

	*N* ^a^	Detection frequency (%)	Median (µg/L)	UWSs exceeding MCL (%)
Uranium				
Navajo Nation	463	75.0	3.8	12.5
*Chinle*	90	67.8	4.0	10.8
*Eastern*	86	48.8	0.4	8.4
*Fort Defiance*	97	70.1	2.0	8.8
*Shiprock*	79	88.6	6.6	17.6
*Western*	111	95.5	5.3	18.5

	*N* ^a^	Detection Frequency (%)	Correlation *τ* (*p* value)	UWSs exceeding ½ both MCLs (%)	UWSs exceeding both MCLs (%)
Arsenic and uranium					
Navajo Nation	464	49.0	0.23 (<0.001)**	8.4	3.9
*Chinle*	90	22.2	0.01 (0.85)	2.2	1.1
*Eastern*	87	40.2	0.33 (<0.001)**	5.7	4.6
*Fort Defiance*	97	60.8	0.34 (<0.001)**	7.2	3.1
*Shiprock*	79	54.4	0.19 (0.01)*	12.7	2.5
*Western*	111	63.1	0.19 (0.002)**	13.5	7.2

* significant at an α level of 0.01; ** significant at an *α* level of < 0.001

^a^ *N* is the total number of water sources

Arsenic and U were detected simultaneously in approximately half of sources throughout Navajo and were positively correlated throughout Navajo, except in Chinle Agency where no significant correlation was observed. Overall, 3.9 % of the sources simultaneously exceeded both the As and U MCL, however the proportion was highest in the Western Agency where more than 7 % of sources exceed both MCLs.

**Fig. 2:**
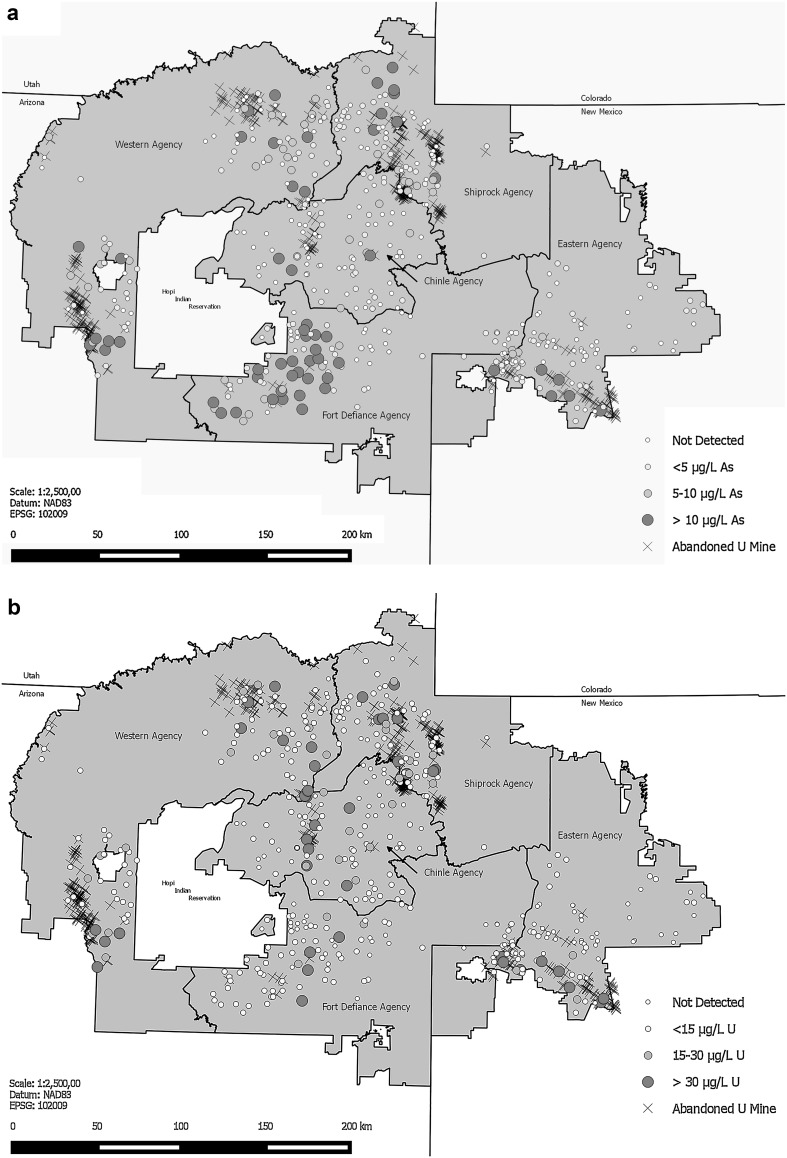
Graduated *dot maps* illustrating the concentration of arsenic (**a**) and uranium (**b**) in unregulated water sources

Using a geographic information system we visually evaluated the geospatial distribution of As and U throughout Navajo. Arsenic was detected in the majority of sources in northern and southern Navajo while fewer sources in central and eastern Navajo had detectable concentrations (Fig. 2a). The majority of MCL exceedances occurred along the central north/south axis of Navajo, with additional exceedances observed in the Cameron area and the Eastern Agency. The largest group of As MCL exceedances was located in south-central Navajo (around Hopi Buttes in Fig. 1). In contrast, U was detected in sources throughout Navajo (Fig. 2b). Elevated U concentrations were observed in Eastern, Shiprock and Western Agencies, irrespective of proximity to an abandoned mine. Compared to As, there was greater overlap between U MCL exceedances and abandoned mine proximity. Uranium MCL exceedances were also observed, however, outside of mining areas. Co-exposure to As and U concentrations greater than half of the MCLs was most common in sources located southwest of the Hopi Reservation, in northern Navajo, and in the Eastern Agency.

One hundred and seventy-five sources were located within 6.4 km of an abandoned U mine (Table 3). Compared to sources located beyond this distance, the closer sources had higher median concentrations of As (*X* ^2^ = 12.3, *p* = 0.0005) and U (*X* ^2^ = 43.1, *p* value < 0.0001), and were 6.3 % more likely to exceed the As MCL and 8.3 % more likely to exceed the U MCL.

**Table 3:** Summary statistics for As and U, partitioned by distance to an abandoned uranium mine (AUM)

UWS	*N* ^a^	Detection frequency (%)	Median (µg/L)	IQR^b^ (µg/L)	UWSs exceeding MCL (%)
Arsenic					
<6.4 km from AUM	176	54.3	3.4	1.1–7.2	20.0
>6.4 km from AUM	288	55.6	1.0	0.4–4.0	13.3

UWS	*N* ^a^	Detection frequency (%)	Median (µg/L)	IQR^b^ (µg/L)	UWSs exceeding MCL (%)
Uranium					
<6.4 km from AUM	175	86.3	7.0	2.1–20.1	17.8
>6.4 km from AUM	288	96.8	2.0	0.3–7.6	9.5

UWS	*N* ^a^	Detection frequency (%)	UWSs exceeding ½ both MCLs (%)	UWSs exceeding both MCLs (%)
Arsenic and Uranium				
<6.4 km from AUM	176	53.4	12.5	5.1
>6.4 km from AUM	288	46.2	5.9	3.1

^a^ *N* is the number of water sources; ^b^ IQR is the interquartile range: 25th percentile to 75th percentile

**Fig. 3:**
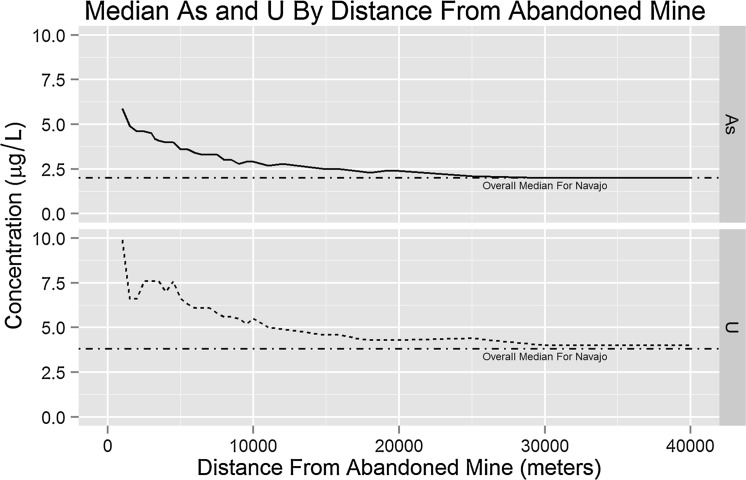
*Line plot* illustrating median As (*upper panel*) and U (*lower panel*) concentration as a function of distance from an abandoned U mine. The *dotted line* in each panel represents the overall median concentration for all tested unregulated sources on Navajo

We also plotted the median As (Fig. 3 upper panel) and U (Fig. 3 lower panel) concentration as a function of distance from an abandoned mine. We observed a declining trend for both As and U concentration as distance increased. The median As concentration reached the overall Navajo median between 25 and 30 km and the median U concentration approached the overall Navajo median between 30 and 40 km. The rates of As and U co-occurrence in UWSs were similar when partitioned at the 6.4 km distance, and these frequencies were comparable to co-occurrence throughout Navajo generally (Table 3).

## Discussion

For Navajo, a large and predominantly rural area, the combination of naturally occurring mineral deposits, historic mining, and limited water infrastructure increases potential exposure to inorganic chemicals via unregulated water sources. Our results suggest that As and U are widely distributed on Navajo, often at concentrations exceeding drinking water standards, and at a frequency similar to other areas of the southwest (Athas 2010; Uhlman et al. 2010) and greater than the United States generally (Ayotte et al. 2011; Lee and Helsel 2005) (Table 4). The co-occurrence of As and U in sources is also more frequent on Navajo than for the United States generally (DeSimone et al. 2009). Arsenic and U concentrations in water sources located within 6 km of an abandoned mine site were more likely to exceed an MCL than sources located further away. Naturally occurring As and U mobilization may contribute to elevated concentrations in these sources because MCL exceedances were observed throughout Navajo regardless of mine proximity. Compiling information from previous geographically focused studies, and collectively analyzing and mapping the results enabled us to synthesize a geospatial understanding of the regional similarities and differences in As and U occurrence. Below we comment on potential influence of abandoned mines on water quality, public policy goals for exposure reduction and increasing public water access, and potential human health impacts of co-exposure to As and U.

**Table 4:** Summary of arsenic and uranium detection and MCL exceedance frequencies

	Arsenic			Uranium			Arsenic and uranium	
	NN	US	Other studies	NN	US	Other studies	NN	US
Detection (%)	55.1	51^a^		75.0	53^a^	68^h^	49.0	
Median (µg/L)	2.0	0.72^b^		3.8	0.52^g^			
>MCL (%)	15.1	6.8–10.6^a,b,c^	10–17^d,e,f^	12.5	1.7–3.7^a,c^	6.3^h^	3.9	0.3^c^

*NN* Navajo Nation, *US* United States

^a^ Focazio et al. (2006); ^b^ Lee and Helsel (2005); ^c^ DeSimone et al. (2009); ^d^ Athas (2010); ^e^ Uhlman et al. (2010); ^f^ Murphy et al. (2009); ^g^ Ayotte et al. (2011); ^h^ Eggers et al. (2015)

### Potential Influence of Abandoned Mines on As and U Groundwater Concentrations

The *natural occurrence* of As and U has been documented in groundwater sources in the Cameron and Monument Valley mining districts of Western Agency (Longsworth 1994), in water sources along the Puerco River in the Eastern Agency (Wirt 1994) and in northern New Mexico (McQuillan and Montes 1998). In contrast, mine waste and spills have been associated with localized contamination of groundwater sources downstream of the 1979 Northeast Church Rock Mine spill (Wirt 1994), mill waste in Shiprock Agency (US DOE 2011), and mill waste in Tuba City and Cane Valley in Western Agency (US DOE 1996; US DOE 1998). In our team’s previous investigations at the Claim #28 mine site in the Central Agency, Blake et al. (2015) determined that U and As mobility in groundwater was influenced by the dissolution and reactivity of uranyl vanadates and the presence of arsenic-iron bearing minerals in ore waste. On the Navajo Nation source attribution of chemicals in groundwater has not been extensively studied.

While there is limited evidence linking historic U mining with widespread contamination of groundwater, we did observe that unregulated sources located closer to abandoned mines more frequently exceeded As and U drinking water standards. We hypothesize this could occur because: (1) unregulated sources draw water from the original ore-bearing geological formation and is therefore naturally occurring; (2) groundwater is contaminated by mine waste; or (3) there are chemical alterations in groundwater introduced by the mining process leading to mobilization of minerals; however, the compiled data are insufficient for evaluating geochemical processes needed to test these hypotheses, which warrants further investigation. Many of the unregulated sources on Navajo lack well logs so it is challenging to determine associations between geological formation, mines, and water quality. Knowledge of the production formation would be insufficient however to distinguish between natural and anthropogenic occurrence because many of the mined formations are also water bearing formations.

Nonetheless, from a public health perspective, the geospatial analysis was useful for visually identifying areas of similar chemical concentrations in unregulated sources so that local communities may be informed of potential risk. Regardless of the cause, natural or anthropogenic, elevated As and U concentrations were observed in unregulated sources in areas throughout the Navajo Nation, especially those near abandoned mine sites. The elevated occurrence frequency is concerning because there are several thousand homes located near abandoned mine sites that lack public water access (Navajo Access Workgroup 2010). If people in these areas haul water from unregulated sources also located near abandoned mines there is a greater potential for As and U exposure. The demonstrated potential for co-exposure to As and U should be useful in prioritizing policy decisions related to infrastructure development and risk reduction strategies to protect public health.

### Implications for Exposure Reduction Strategies

The spatial analysis of As and U occurrence illustrated areas of elevated occurrence that coincide with previous work identifying areas with limited public water system infrastructure, such as areas of Fort Defiance, Western and Shiprock Agencies. There are several exposure reduction strategies that could be employed including expanding water infrastructure, and point-of-use filters. Expanding water infrastructure to increase the number of homes connected to a public water system is likely expensive and impractical in some areas due to the low population density and large distances between households in rural Navajo. A second option is to selectively expand infrastructure to provide regulated water hauling stations. There are currently 67 water hauling stations spread throughout Navajo but there are some areas with few hauling stations and a large number of homes without public water access. In areas where As and U occurrence is common, public water systems are not widely accessible, and existing water hauling stations are too far for residents to use regularly, establishing additional hauling stations could increase access to regulated drinking water.

Because of the significant expense of infrastructure expansion and low population density of Navajo, point-of-use risk reduction strategies, such as filtration, may be more appropriate in some situations. The selected filtration method will depend on water chemistry for individual sources, but may be informed by regional knowledge of As and U occurrence. For example, sources in Central and Eastern Agencies tend to have low concentrations of As but higher U, so filtration in these areas could emphasize U removal; whereas in Fort Defiance Agency, As is more common and filtration efforts could prioritize As reduction. In Western and Shiprock Agencies, and sources near abandoned mine sites, As *and* U concentrations tend to be elevated suggesting the need for a hybrid removal system. Evaluation of exposure reduction strategies should also consider social challenges such as poverty that may make point-of-use filtration systems economically impractical for some households.

### Human Health Implications of As and U Exposure

Individually, As and U are known to impact human health. Chronic exposure to As is associated with neuropathy, developmental disabilities, decreased IQ, numerous skin disorders, hypertension, and cancer of the skin, lungs, bladder and kidney (Abernathy et al. 2003; Buchet and Lison 2000; Kapaj et al. 2006; Kavcar et al. 2009). Exposure to U through drinking water is another public health concern because U is a known nephrotoxicant (Kurttio et al. 2006; Vicente–Vicente et al. 2010). It is also a bone seeking chemical (Kurttio et al. 2004) that can cause genotoxicity and developmental defects (Brugge and Buchner 2011) and bioaccumulate in bone (Kurttio et al. 2004).

The frequency of co-occurring exceedances of As and U presents a potential public health risk for unregulated water source users on Navajo, and more generally for populations living in other mining areas in the western United States, since few studies have examined the health impacts associated with this chemical mixture. Ongoing studies by our group have found a positive association between intake of As in drinking water and oxidized low-density lipoprotein (oxLDL), a novel biomarker of cardiovascular disease, among a Navajo cohort in the Eastern Agency (Harmon 2016). In contrast, consumption of U in drinking water reduced the concentration of oxLDL, suggesting a need to investigate chemical interactions occurring with co-exposure to predict health outcomes. In the same Navajo cohort we also observed higher rates of self-reported kidney disease among people who were exposed to As, U and other chemicals in U mine wastes (Hund et al. 2015). Current uncertainty about the health impacts of co-exposure to As and U, and documented co-occurrence in unregulated sources in rural, former mining areas with known health disparities indicates a need to better understand the toxicology of this chemical mixture and a need for intervention strategies that reduce co-exposure. While the health outcomes of As and U co-exposure remain uncertain, there is potential for synergistic toxicological interaction of these chemicals as some common mechanisms of action have been found (Cooper et al. 2016).

### Limitations

The water quality data collected for the set of sources presented in this study were not collected to evaluate geochemical processes influencing mobilization of As or U in groundwater or the impact of abandoned mines on groundwater quality; these data were collected to evaluate potential exposure via ingestion of water from these sources. Additionally, as illustrated in Fig. 2, there are several areas of Navajo where no water quality results were available. Therefore, data aggregated to the management agency level may not adequately represent the water quality of sub-units within each agency. This limitation is of particular important for the Cameron area of southwest Navajo where very little information about unregulated source quality was available and a significant number of homes lack access to a public water supply (Navajo Access Workgroup 2010). Future efforts should work with communities in areas with limited sampling and known public water access challenges to evaluate inorganic chemical occurrence, estimate metals intake via unregulated water sources, and explore exposure reduction methods.

Most of the unregulated sources evaluated in this study were sampled one time and as a result temporal variability was not evaluated. For sources with multiple samples, the average standard deviation for As and U was 0.48 and 1.4 µg/L respectively. Water sources with multiple samples had a correlation coefficient (*τ*) of 0.499 for As and 0.599 for U. Only 1.7 % of sources transitioned from not exceeding to exceeding the MCL when the minimum and maximum observations were compared. This analysis was limited to As and U only, though the occurrence of other chemicals is possible in these water sources. We determined that beryllium, cadmium, chromium, nickel and lead were detected in less than 20 % of sources and that concentrations of barium, copper, manganese, selenium and zinc exceeded regulatory (i.e., MCL) or health-based screen levels (Toccalino and Norman 2006) in fewer than 5 % of unregulated sources. Although a water source may contain low concentrations of As, U or other inorganic chemicals, it does not indicate that the source is free of other toxicants such as microorganisms, which can cause acute health effects. Because we did not include organic contaminants, pesticides, bacteria, or radionuclides, it should not be concluded that As and U present the only health risk for unregulated sources (Toccalino et al. 2012).

## Conclusions

In regions with limited water infrastructure and mining history, consumption of water from unregulated sources may contribute to health disparities observed among rural and tribal populations. On Navajo, approximately 30 % of households lack access to a public water system and may consume water from an unregulated source. The results of our study illustrate the regional spatial variability of As and U occurrence and areas of elevated chemical concentrations. Collectively these results inform remediation strategies and can help shape public policy goals for providing public water access to Navajo residents. Our findings reinforce the need for water quality testing for multiple inorganic contaminants, a need to continue addressing rural water challenges in areas potentially impacted by abandoned mines, and continue efforts to educate people about the challenges and potential health risks of consuming unregulated water.

## Abbreviations

As: Arsenic
U: Uranium
UWSs: Unregulated water sources
